# Embryonic periventricular endothelial cells demonstrate a unique pro-neurodevelopment and anti-inflammatory gene signature

**DOI:** 10.1038/s41598-020-77297-3

**Published:** 2020-11-23

**Authors:** Franciele Cristina Kipper, Cleide Angolano, Ravi Vissapragada, Mauricio A. Contreras, Justin Moore, Manoj Bhasin, Christiane Ferran, Ajith J. Thomas

**Affiliations:** 1 Division of Neurosurgery, Beth Israel Deaconess Medical Center, Boston, MA 02215 USA; 2 Division of Vascular and Endovascular Surgery, Department of Surgery, Beth Israel Deaconess Medical Center, Boston, MA 02215 USA; 3 Division of Vascular Surgery, Department of Surgery, Beth Israel Deaconess Medical Center, Boston, MA 02215 USA; 4 Discipline of Surgery, College of Medicine and Public Health, Flinders University, Adelaide, 5042 SA Australia; 5 Center for Vascular Biology Research, Beth Israel Deaconess Medical Center, Boston, MA 02215 USA; 6 BIDMC Genomics, Proteomics, Bioinformatics and Systems Biology Center, Beth Israel Deaconess Medical Center, Boston, MA 02215 USA; 7 Harvard Medical School, Boston, MA 02215 USA

**Keywords:** Angiogenesis, Neurogenesis, Stem-cell niche

## Abstract

Brain embryonic periventricular endothelial cells (PVEC) crosstalk with neural progenitor cells (NPC) promoting mutual proliferation, formation of tubular-like structures in the former and maintenance of stemness in the latter. To better characterize this interaction, we conducted a comparative transcriptome analysis of mouse PVEC vs. adult brain endothelial cells (ABEC) in mono-culture or NPC co-culture. We identified > 6000 differentially expressed genes (DEG), regardless of culture condition. PVEC exhibited a 30-fold greater response to NPC than ABEC (411 vs. 13 DEG). Gene Ontology (GO) analysis of DEG that were higher or lower in PVEC vs. ABEC identified “Nervous system development” and “Response to Stress” as the top significantly different biological process, respectively. Enrichment in canonical pathways included HIF1A, FGF/stemness, WNT signaling, interferon signaling and complement. Solute carriers (SLC) and ABC transporters represented an important subset of DEG, underscoring PVEC’s implication in blood–brain barrier formation and maintenance of nutrient-rich/non-toxic environment. Our work characterizes the gene signature of PVEC and their important partnership with NPC, underpinning their unique role in maintaining a healthy neurovascular niche, and in supporting brain development. This information may pave the way for additional studies to explore their therapeutic potential in neuro-degenerative diseases, such as Alzheimer’s and Parkinson’s disease.

## Introduction

The development of the central nervous system (CNS) involves close orchestration between neural and vascular components^1^. During embryogenesis, neural tube development (~ E7.5–E9.5 in mouse) precedes and promotes the formation of circumscribing perineural or pial vascular plexuses^2^, which give rise to brain vessels. Starting at E9.5, vascularization of the mouse neural tube results in the formation of periventricular vessels, originating from the telencephalic floor of the basal ganglia primordium, which in turn give rise to the embryonic forebrain arterial network by E11.5. Neurogenesis and angiogenesis in the embryonic brain are tightly coupled processes^2–4^.

Studies elucidating the role and function of the brain neurovascular unit (NVU) in health and disease have resulted in a paradigm shift from an exclusively “neurocentric” perspective to a more integrative process that emphasizes the dynamic cross-talks within the NVU between endothelial cells (EC), neurons, astrocytes, pericytes, and microglia^4–7^. EC-regulated self-renewal and proliferation of neural progenitor cells (NPC) was observed in cerebral vascular niches^7,8^. The NVU also regulates local blood flow and integrity of the blood–brain barrier (BBB) via intricate signaling between its different components^9–11^. Prior studies exploring the symbiotic modulation of neurogenesis and angiogenesis in the context of neurodegeneration were primarily based on adult brain endothelial cells (ABEC), and hence failed to explore the potentially superior, and certainly more physiologic ability of embryonic brain EC to influence neuroangiogenesis^12,13^. We previously showed that embryonic brain periventricular EC (PVEC) surpass ABEC ability to promote NPC proliferation while delaying its differentiation^4^, although the identity and role of PVEC remained poorly understood. To address this gap, we performed a comparative RNA-seq based global gene expression analysis of PVEC vs. ABEC at baseline, and after co-culture with NPC. Our results show that ABEC and PVEC have a dramatically different transcriptome regardless of culture condition. At baseline, “transcription targets of Hypoxia Inducible Factor (HIF)-1” was the most modified pathway, with “WNT signaling” and “Interferon α/β signaling via JAK/STAT” being the most positively and negatively impacted pathways, respectively. Notably, solute carriers (SLC) and ABC transporters represented an important subset of differentially expressed genes (DEG), underscoring PVEC’s potential role in BBB formation. Our results also strongly demonstrate that PVEC were significantly more prone to transcriptional changes than ABEC when co-cultured with NPC, further highlighting their prominent role in shaping the NVU during early embryonic brain development. Specifically, complement and immune response pathways were the most downregulated in PVEC following NPC co-culture, supporting the anti-inflammatory phenotype of these cells.

## Results

### Baseline gene expression profile shows major differences between PVEC and ABEC

**Figure 1:**
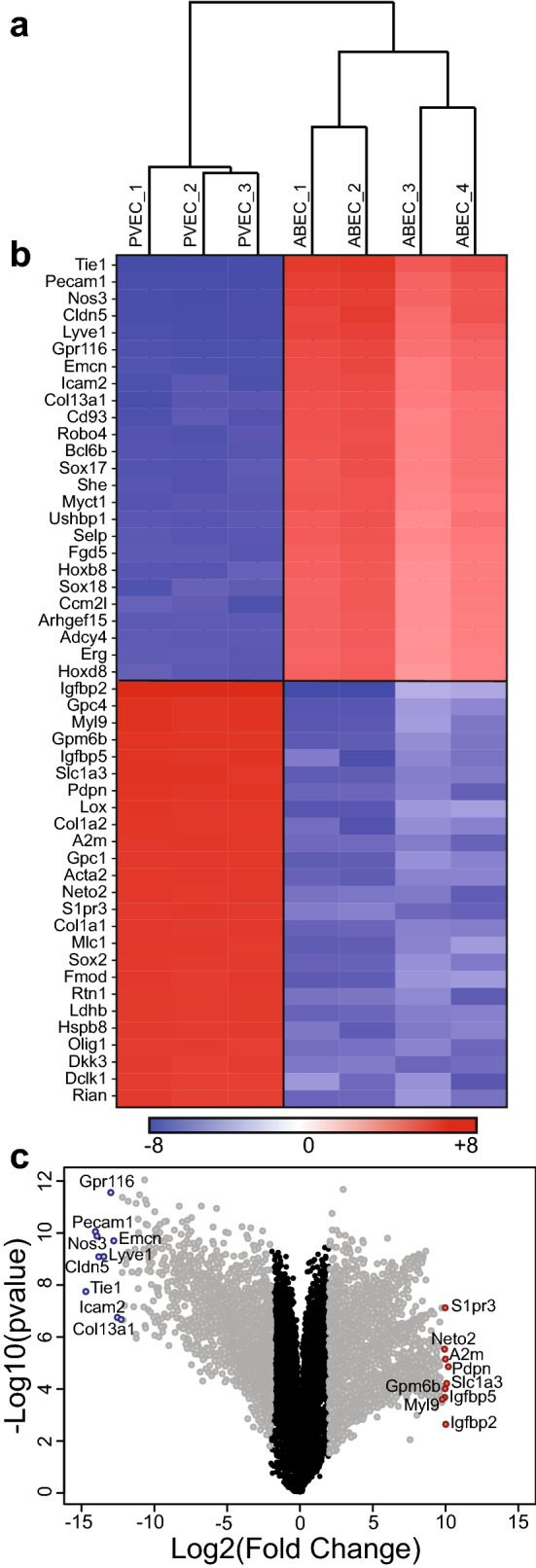
Baseline expression profile of PVEC and ABEC highlights the distinct identity of these vascular beds. (**a**) Hierarchical clustering indicates total segregation between PVEC and ABEC. (**b**) Heatmap of the top 50 genes that were significantly different in PVEC vs. ABEC, based on fold change. Red indicates genes with higher expression and blue indicates genes with lower expression in PVEC vs. ABEC. (**c**) Volcano plot encompassing all analyzed genes, depicted as dots. Black dots represent genes that were not significantly different, while gray dots represent genes that were significantly different in PVEC vs. ABEC, based on fold change (FC) > 2 and p-value < 0.05. Highlighted in blue dots are the genes that were most significantly lower, and in red dots genes that were most significantly higher in PVEC vs. ABEC.

PVEC were isolated from E15.5 mouse embryos, cultured until confluence, then sorted by CD31-coated magnetic beads, and characterized, as described^4^. PVEC and ABEC were cultured for 48 h prior to RNA extraction and subsequent RNAseq analysis. Each sample interrogation yielded 25–30 million reads. A particular gene was considered differentially expressed (DEG) if the 90% lower confidence bound of the fold change (FC) between samples was > 2. Hierarchical clustering, using Pearson correlation distance metric on unsupervised RNAseq data showed perfect segregation based on EC bed (Fig. 1a).

The top 25 genes that were significantly higher in PVEC vs. ABEC included podoplanin (*Pdpn*), solute carrier (Slc) family 1 member 3 (*Slc1a3*), α-2-macroglobulin (*A2m*), membrane glycoprotein 6B (*Gpm6b*), insulin-like growth factor binding protein 5 (*Igfbp5*), neuropilin and tolloid like 2 (*Neto2*), myosin light chain 9 (*Myl9*) and glypican 4 (*Gpc4*) (Fig. 1b). Based on the literature, most of these genes reflect PVEC stemness and their ability to promote neural development^14–16^. The top 25 genes that were significantly lower in PVEC vs. ABEC included a number of EC-specific or EC-enriched molecules such as tyrosine kinase with immunoglobulin-like and EGF-like domains 1 (*Tie1*), platelet/endothelial cell adhesion molecule 1 (*Pecam1*), nitric oxide synthase 3 (*Nos3*), claudin 5 (*Cldn5*), lymphatic vessel endothelial hyaluronan receptor 1 (*Lyve1*), adhesion G-protein coupled receptor F5 (*Adgrf5/Gpr116*) and endomucin (*Emcn*) (Fig. 1b). These markers are highly expressed in mature adult brain EC^17^, hence their lower expression in PVEC implies the relative immaturity of these cells. Although ABEC and PVEC share a common endothelial identity and brain origin, a supervised analysis identified a staggering 5842 (2673 higher and 3169 lower) DEG between them, as depicted in the Volcano plot (Fig. 1c).

### The PVEC transcriptome is enriched in genes that positively promote nervous system development and dampen inflammatory responses

**Figure 2:**
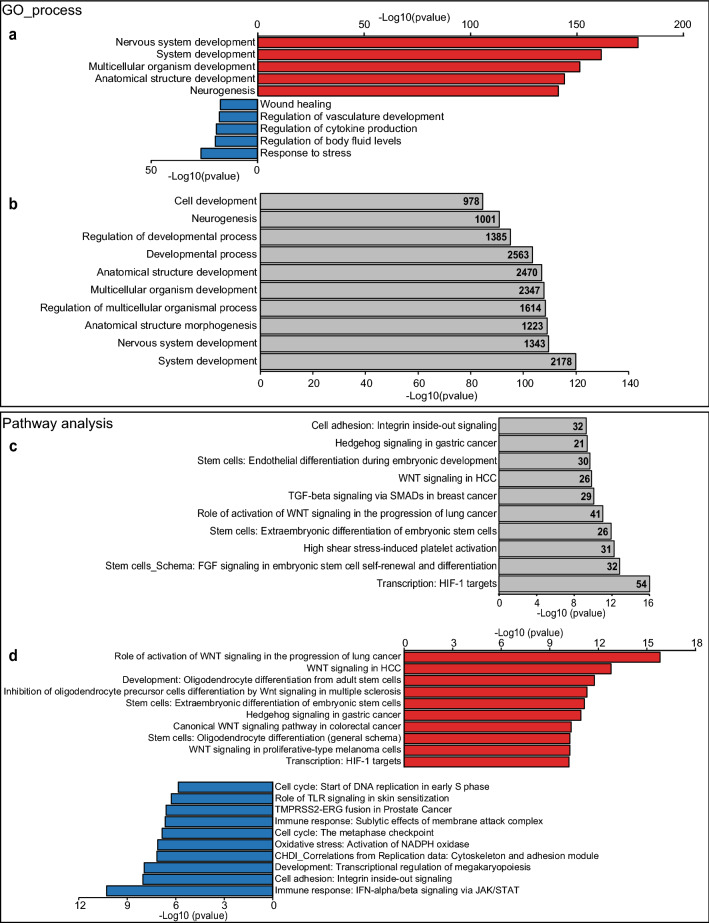
GO analysis identifies nervous system development and response to stress as the processes with the greatest number of DEG that were higher or lower in PVEC vs. ABEC. (**a**) Gene Ontology (GO) analysis using the Metacore from Clarivate Analytics identified the top 5 most enriched biological process when separated by higher (red) or lower (blue) differentially expressed genes (DEG) in PVEC vs. ABEC. (**b**) The top 10 more enriched biological processes when all DEG were analyzed together closely resembles the analysis performed using DEG that were higher in PVEC vs. ABEC. Numbers inside the bars correspond to the number of DEG in each given GO biological process. (**c**) Canonical pathway enrichment analysis using the Metacore identified the top 10 most enriched pathways in PVEC vs. ABEC, here depicted as grey bars. Numbers inside the bars correspond to the number of DEG that were identified in each given pathway. (**d**) Canonical pathway enrichment using the Metacore, following partitioning of our data into genes that were higher (red) or lower (blue) in PVEC vs. ABEC identified the top 10 pathways in each category.

We performed Gene Ontology biological process (GO_bp) analysis of DEG using Metacore from Clarivate Analytics. When analyzing transcripts that were higher in PVEC vs. ABEC, as expected, processes such as nervous system development, neurogenesis, generation of neurons, developmental processes and neuron differentiation were recognized among the top 5 most significant GO_bp. Analysis of transcripts that were lower in PVEC vs. ABEC identified processes such as response to stress, regulation of cytokine production and wound healing among the top 5 most significant GO_bp (Fig. 2a). In the combined datasets, the top 10 GO_bp were mostly related to those identified when using the “only higher” dataset, more specifically system development and nervous system development (Fig. 2b).

Canonical pathway enrichment also using the Metacore software identified pathways enriched in PVEC vs. ABEC. The top 3 most enriched pathways include “Transcription targets of Hypoxia Inducible Factor 1” (HIF-1), with hypoxia inducible factor 1, alpha subunit (*Hif1a*) transcript levels being twofold lower in PVEC vs. ABEC, “Stem cells Schema: FGF signaling in embryonic stem cell renewal and differentiation”, and “High shear stress platelet activation” (Fig. 2c). More than half of the genes in the HIF-1 pathway (54 of 95) were differentially expressed in PVEC vs. ABEC. Of these, 33/95 were > twofold higher, and 21/95 were > twofold lower (Table 1 and Supplementary Figure 2a). Specifically, transcript levels of: (a) angiogenesis-related vascular endothelial growth factor A (*Vegfa*, tenfold), fibroblast growth factor 2 (*Fgf2*, 2.3-fold), plasminogen activator inhibitor (PAI1/S*erpine1*, fourfold), thrombospondin 1 (*Thbs1*, 4.4-fold), and angiopoietin 2 (*Angpt2*, 4.8-fold) were higher, while those of chemokine (C-X-C motif) ligand 12 (*Cxcl12/Sdf1*, 14-fold), FMS-like tyrosine kinase 1 (*Flt1/Vegfr1*, 11-fold), endoglin (*Eng*, 13.7-fold), mast cell growth factor (*Mgf/Kitl*, 8.4-fold) and placental growth factor (*Pgf/Plgf*, 8.5-fold) were lower; (b) regulators of extracellular matrix (ECM) remodeling matrix metallopeptidase 2 (*Mmp2*, 83.5-fold), lysyl oxidase-like 2 (*Loxl2*, 20.8-fold), and lysyl oxidase (*Lox*, 639-fold) were higher, while those of plasminogen activator urokinase receptor (*Plaur*, 3.4-fold), *Loxl4* (18-fold) were lower; (c) regulators of glucose metabolism *Slc2a3/Glut3* (421-fold), hexokinase 2 (*Hk2*, 16.5-fold) and *Slc16a3/Mct4* (twofold) were higher, while hexokinase 1 (*Hk1*, 2.3-fold), enolase 1 (*Eno1*, 904-fold), phosphofructokinase, liver, B-type (*Pfkl*, 2.4-fold), and phosphoglycerate kinase 1 (*Pgk1*, 2.4-fold) were lower; (d) maintenance of stemness sex determining region Y (SRY)-box 2 (*Sox2*, 708-fold) was higher, while nanog homeobox (*Nanog*, ninefold) and POU domain, class 5, transcription factor 1 (*Pou5f1/Oct-3/4*, ninefold) were lower; (e) chemotaxis-associated chemokine (C-X3-C motif) receptor 1 (*Cx3cr1*, threefold) and *Cxcr4* (401-fold) were higher, and (f) fibrosis/immunomodulatory cellular communication network factor 2 (*Ccn2/Ctgf,* 16-fold), transforming growth factor beta 2 (*Tgfb2,* ninefold) and *Tgfb3* (33-fold) were higher, while *Tgfb1* (threefold) was lower.

**Table 1:** Differentially expressed genes in the “transcription: HIF-1 targets” pathway.

Process	Gene	Fold change	p-value
Angiogenesis	VEGF-A	10.14	1.15E−11
	α1B adrenergic receptor	7.34	5.64E−05
	Angiopoietin 2	4.79	3.73E−09
	Thrombospondin 1	4.40	6.40E−08
	PAI	4.09	9.08E−08
	Adrenomedullin	2.71	1.01E−07
	FGF2	2.32	4.14E−02
	MGF	− 8.36	1.70E−10
	PLGF	− 8.52	1.77E−09
	VEGFR-1	− 11.05	2.45E−09
	Endoglin	− 13.70	2.62E−11
	SDF-1	− 14.11	1.21E−11
Glucose metabolism	GLUT3	421.30	1.27E−06
	ALDOC	74.49	6.35E−09
	HXK2	16.55	4.62E−09
	F263	2.87	7.85E−08
	MCT4	2.00	1.35E−04
	HXK1	− 2.32	9.61E−09
	PGK1	− 2.43	2.08E−07
	PFKL	− 2.43	3.17E−09
	PDK1	− 2.45	2.25E−07
	ENO1	− 903.94	2.56E−13
Extracellular matrix remodeling	Lysyl oxidase	638.91	7.68E−05
	MMP-2	83.05	9.41E−10
	LOXL2	20.82	4.43E−11
	P4HA2	− 2.11	3.84E−08
	PLAUR	− 3.36	1.31E−08
	LOXL4	− 18.11	6.34E−09
Fibrosis immuno-regulation	TGF-beta3	33.51	7.20E−11
	CTGF	15.95	1.36E−09
	TGF-beta2	9.03	1.27E−08
	TGF-beta1	− 3.38	1.34E−08
Stem cell maintenance	SOX2	708.34	6.72E−06
	Oct-3/4	− 9.17	6.98E−04
	NANOG	− 9.57	8.78E−08
Cell cycle arrest	p21	2.10	1.15E−08
	c-Myc	-2.01	3.59E−07
Chemotaxis	CXCR4	400.55	3.39E−09
	CX3CR1	3.17	1.35E−02
Transcription regulation	ROR-alpha	100.74	1.49E−08
	ID2	2.31	1.84E−08
Nucleotide metabolism	AK3	3.84	1.09E−07
	Nt5e	− 2.03	7.39E−05
Iron ion metabolism	Ceruloplasmin	80.37	9.94E−12
	Transferrin	61.58	4.14E−08
Regulation of pH	Carbonic anhydrase IX	11.86	2.17E−04
Lipid metabolism	A2M/LRP1	10.40	2.67E−08
Amino acid metabolism	TGM2	− 3.85	3.99E−08
Negative regulation of HIF1A signaling	CITED2	3.61	5.16E−10
Regulation of cell proliferation	IBP3	16.22	1.35E−10
Apoptosis	NOXA	729.95	7.75E−08
Heme metabolism	FECH	2.42	5.43E−09
Other	MDR1	− 124.65	1.32E−11

List of transcripts DEG sorted by process and fold change, with respective p-value when compared PVEC vs. ABEC.

A substantial number of genes (32 of 47) included in the “FGF signaling in embryonic stem cell self-renewal and differentiation” pathway were differentially expressed in PVEC vs. ABEC. Amongst those, higher levels of genes encoding for secreted proteins noggin (*Nog*, 153-fold), insulin growth factor 2 (*Igf2*, 47-fold), gremlin 1 (*Grem1*, 30-fold) and *Fgf2* predict PVEC propensity to inhibit Bone Morphogenetic Protein (BMP) signaling, and favor the maintenance of pluripotency and stemness in both neural and hematopoietic lineages^18^. Nonetheless, dramatically lower levels of the *Sox17* transcript (4222-fold) in PVEC likely precludes their transition into hematopoietic stem cells, thereby maintaining their commitment to the EC lineage^19^. Also, 31 of 46 genes associating with the “High shear stress-induced platelet activation” pathway were differentially expressed in PVEC vs. ABEC. There were significantly lower transcript levels of Von Willebrand factor (*Vwf*, 1066-fold), integrin beta 3 (*Itgb3*, 14-fold), and selectin platelet (*Selp/*P-selectin, 3556-fold), all of which were consistent with an immature, yet less thrombogenic and less inflammatory PVEC phenotype^20^.

Next, we analyzed canonical pathways after separating the dataset by DEG that were higher or lower in PVEC vs. ABEC. This identified the “role of activation of WNT signaling in progression of lung cancer” as the pathway with the greatest number of DEG whose expression was higher (36/77) in PVEC, and “immune response IFNα/β signaling via JAK/STAT” as the pathway with the greatest number of DEG whose expression was lower (25/64) in PVEC (Fig. 2d). Most of the DEG in the Wingless-Type MMTV Integration Site (WNT) pathway were increased by multiple fold (Table 2 and Supplementary Figure 2b). This included 10 of the WNT genes themselves (*Wnt7a*, 69-fold) as well as frizzled ligands of WNT, i.e. secreted frizzled-related protein 1 (*Sfrp1*, 500-fold) and frizzled class receptors (*Fzd1*, 600-fold; *Fzd3*, 300-fold). This also included transcripts of the WNT complex associated proteins axin 1 (*Axin1*, 167-fold), Disheveled family members *Dvl1*, *Dvl2* and *Dvl3* (2–threefold), neuroprotective dickkopf WNT signaling pathway inhibitor 3 (*Dkk3*, 853-fold), and achaete-scute family bHLH transcription factor 1 (*Ascl1/*hASH1, 175-fold), a transcriptional regulator of many genes involved in neural development and differentiation. PVEC had also significantly higher transcript levels of lymphoid enhancer binding factor 1 (*Lef-1*, 64-fold), suggesting the engagement of β-catenin, downstream of WNT.

**Table 2:** Differentially expressed genes involved in the WNT signaling pathway.

Family	Gene	Fold change	p-value
Wingless-type MMTV integration site family	Wnt7a	69.05	9.66E−08
	Wnt8b	22.64	2.65E−08
	Wnt2b	13.46	3.66E−08
	Wnt2b	13.46	3.66E−08
	Wnt7b	12.13	5.17E−07
	Wnt3	11.21	3.83E−08
	Wnt3	11.21	3.83E−08
	Wnt2	11.21	6.94E−05
	Wnt2	11.21	6.94E−05
	Wnt9a	8.16	4.57E−10
	Wnt9a	8.16	4.57E−10
	Wnt5b	7.15	2.02E−07
	Wnt5a	5.34	5.90E−10
	Wnt5a	5.34	5.90E−10
	Wnt4	2.37	6.04E−06
	Wnt4	2.37	6.04E−06
Frizzled	Fzd1	599.47	6.89E−07
	Fzd3	226.56	1.75E−08
	Fzd2/10	78.70	1.80E−10
	Fzd7	3.05	1.23E−07
	Fzd8	2.69	5.28E−08
	Fzd9	− 14.35	6.01E−10
	Fzd4	− 18.05	7.14E−11
Tcf(Lef)	Lef1	63.82	2.17E−08
	Tcf7	17.06	7.81E−09
	Tcf7l2	2.03	7.89E−09
	Tcf7l2	2.03	7.89E−09
p38 MAPK	Mapk13	25.61	1.40E−05
	Mapk11	− 8.81	3.56E−09
	Mapk12	− 16.20	7.54E−11
Dishevelled segment polarity protein (DSH)	Dvl3	2.95	1.08E−08
	Dvl2	2.23	1.52E−07
	Dvl1	2.01	4.97E−07
Secreted frizzled-related protein	Sfrp1	501.83	3.66E−08
	Sfrp2	5.34	1.21E−06
Dickkopf WNT signaling pathway inhibitor	Dkk3	853.00	1.07E−09
Axin2	Axin2	166.70	1.30E−08
BMI-1	Bmi1	− 2.35	1.46E−08
Inhibitor of growth family	Ing4	2.21	5.33E−08
Other	hASH1	175.40	3.60E−10
	NKD1	117.22	9.19E−07
	ROR2	100.74	1.49E−08
	WIF1	71.26	5.31E−08
	NOTCH3	70.10	1.21E−08
	VEGF-A	10.14	1.15E−11
	Porcn	8.42	1.70E−07
	Krm1	2.56	1.39E−08
	p21	2.10	1.15E−08
	c-Myc	− 2.01	3.59E−07
	Survivin	− 2.38	4.90E−08
	Oct-3/4	− 9.17	6.98E−04

List of transcripts DEG in the WNT pathway sorted by family/function, and its respective fold change and p-value when compared PVEC vs. ABEC.

Genes associated with the Interferon α and β (IFNα/β) Janus kinase/Signal Transducer and Activator of Transcription factors (JAK/STAT) signaling were significantly lower in PVEC vs. ABEC. These included signal transducer and activator of transcription (Stat) 4 (78-fold), *Stat6* (eightfold), and *Stat5* (fourfold), as well as the 2 key transducers of type I and type II IFN signaling, namely *Stat1* (fourfold) and *Stat2* (fourfold). Reduced expression of STAT1 is likely to uphold proliferative responses and stemness of neural progenitors of the subventricular (SVZ) niche^21^. Decreased IFN II signaling in PVEC was confirmed by lower transcript levels of STAT1 and STAT2 transcriptional targets, XIAP associated factor-1 (*Xaf1*, 232-fold), ubiquitin specific peptidase 18 (*Usp18*, 68-fold), interferon regulatory factor 7 (*Irf7*, 40-fold), caspase-8 (*Casp8*, fourfold), and interferon-induced protein with tetratricopeptide repeats 3 (*Ifit3*/RIG-G, threefold)^22–24^ (Supplementary Figure 2c).

### The expression profile of SLC and ABC transporters underscores differences in nutrient and metabolite transport between PVEC and ABEC

We checked whether expression levels of SLC differ between PVEC and ABEC. In mice, this family of membrane proteins includes 392 members that participate in brain development and in the formation and maintenance of the BBB^25^. Our results showed that 124 SLC transcripts were differentially expressed (61 higher and 63 lower) in PVEC vs. ABEC (Supplementary Figure 3a). Nineteen of the 61 that were higher in PVEC vs. ABEC increased by > 70-fold (Table 3), including the glutamate transporters Slc1a3 (*Eaat1/Glast1*, 1466-fold), Slc1a2 (*Eaat3*, 70-fold), and Slc38a3 (*Snat3*, 131-fold)^26^, the mitochondrial ATP-Mg/Pi carrier *Slc25a23* (237-fold) that protects neurons from glutamate toxicity^27^ and the zinc transporter *Slc39a12* (141-fold), whose function is to support nervous system development^28^. This list also includes hormone transporters, such as *Slcolc1* (*Oatp14,* 654-fold) and *Slc16a2* (*Mct8,* 247-fold), which are involved in thyroid hormone (T4) transport and uptake in brain EC and neurons^29^, and the glucose transporters *Slc2a3* (*Glut3,* 489-fold) and *Slc2a13* (74-fold) that function to promote neural development and whose expression is enriched in the brain^30^. The relevance of the 63 SLC that were lower in PVEC vs. ABEC, including *Slc9b1, Slc24a1, Slc46a3, Slc16a11, Slc6a3* (> 70-fold lower), is not totally clear and needs to be explored (Table 3).

**Table 3:** Differentially expressed SLC transporters in PVEC vs. ABEC.

Gene	Fold change	Protein name	Substrates	Family
Slc1a3	1465.89	EAAC1, EAAT1	L-Glu, D/L-Asp	High-affinity glutamate and neutral amino acid transporter
Slco1c1	654.22	OATP1C1	T4, T3, rT3	Organic anion transporter
Slc2a3	489.48	GLUT3	Glucose, galactose, mannose, xylose	Facilitative GLUT transporter
Slc4a3	295.16	AE3	Chloride bicarbonate	Bicarbonate transporter
Slc6a15	280.17	NTT73	Large, neutral amino acids	Sodium- and chloride-dependent neurotransmitter transporter
Slc16a9	247.31	MCT9		Monocarboxylate transporter
Slc25a23	236.53	APC2	ATP-Mg^2+^, ATP, ADP, AMP, Pi	Mitochondrial carrier
Slco1a5	170.64	OATP-3	Taurocholate and thyroid hormones	Organic anion transporter
Slc39a12	140.97	ZIP12j, LZT-Hs8	Zn	Metal ion transporter
Slc16a2	131.42	MCT8	T2, rT3, T3, T4	Monocarboxylate transporter
Slc38a3	130.64	SNAT3	Q, H, A, N	System A and System N sodium-coupled neutral amino acid transporter
Slc4a4	108.59	NBCe1	Sodium bicarbonate (and/or carbonate)	Bicarbonate transporter
Slc7a2	107.45	CAT-2 (A or B)	Cationic l-amino acids	Cationic amino acid transporter/glycoprotein-associated
Slc15a2	98.45	PEPT2	Di- and tri-peptides, protons, beta-lactam antibiotics	Proton oligopeptide cotransporter
Slc6a1	76.36	GAT-1	GABA	Sodium- and chloride-dependent neurotransmitter transporter
Slc6a17	73.98	NTT4	Neutral amino acids	Sodium- and chloride-dependent neurotransmitter transporter
Slc2a13	73.70	HMIT	Myo-inositol	Facilitative GLUT transporter
Slc1a2	69.88	EAAC1, EAAT3	L-Glu, D/L-Asp	High-affinity glutamate and neutral amino acid transporter
Slc24a3	69.45	Na^+^/K^+^/Ca^2+^-exchange protein 3		Na^+^/(Ca^2+^-K^+^) exchange
Slc22a18	0.0154	ORCTL-2	Probably organic anions	Organic cation/anion/zwitterion transporter
Slc6a3	0.0124	DAT	Dopamine	Sodium- and chloride-dependent neurotransmitter transporter
Slc16a11	0.0106	MCT11	Pyruvate	Monocarboxylate transporter
Slc46a3	0.0102		Lysosomal export of maytansine conjugates	Folate transporter family
Slc24a1	0.0096	NCKX1	Na^+^, Ca^2+^, K^+^	Na^+^/(Ca^2+^-K^+^) exchange
Slc9b1	0.0015	NHE1	Na^+^, Li^+^, H^+^, NH^4+^	Na^+^/H^+^ exchanger

List of SLC family transcripts whose expression was at least 70-fold higher or lower in PVEC vs. ABEC.

Another well characterized superfamily of transporters in brain EC is the ATP-binding cassette (ABC) family, whose members participate in the BBB and the blood CSF barrier^31^. Eighteen out of 52 ABC transporters were differentially expressed in PVEC vs. ABEC (Supplementary Figure 3b). Nine of those were significantly higher, including *Abca9* (96-fold), *Abca8a* (eightfold), *Abca8b* (32-fold) and *Abcg4* (75-fold) that contribute to the transport and metabolism of lipid and cholesterol^32^. The other nine were significantly lower, including membrane P-glycoprotein (*Abcb1a*, 125-fold and *Abcb1b*, sevenfold) whose expression at the luminal site of the BBB increases with gestational age to block the diffusion into the developing brain of deleterious substances and xenobiotics, marking the immature status of the BBB in PVEC^33^.

### PVEC undergo a major transcriptome change upon co-culture with NPC

**Figure 3:**
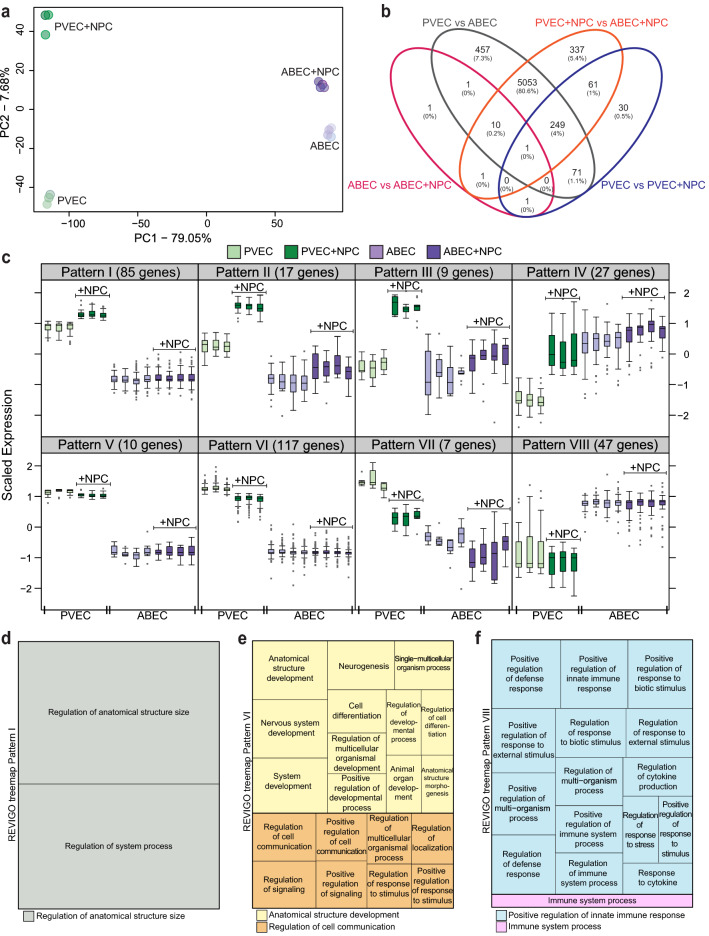
Comparative transcriptomic analysis of PVEC vs. ABEC following co-culture with NPC indicate major changes in PVEC not ABEC. (**a**) Principal component analysis (PCA) of transcriptome. Colors of circles refer to the origin of samples: green PVEC and purple ABEC, light versus dark shades indicate mono vs. NPC co-culture conditions. (**b**) Venn Diagram of DEG in PVEC vs. ABEC (grey ellipse), PVEC + NPC vs. ABEC + NPC (orange ellipse), ABEC vs. ABEC + NPC (pink ellipse) and PVEC vs. PVEC + NPC (blue ellipse). (**c**) Eight different self-organizing maps (SOM) patterns of gene expression profiles were identified amongst the 321 genes that were different in PVEC vs. PVEC + NPC and also in PVEC vs. ABEC. The upper panels display patterns whose gene expression is upregulated in PVEC + NPC as compared to baseline PVEC. Data is shown as standard boxplots (box and whisker plots), depicting the median bar and the dots representing the outliers. Lower panels depict patterns whose gene expression is downregulated in PVEC + NPC as compared to baseline PVEC. Treemap representation of GO_bp (biological process) using REVIGO (reduced visualization of GO) corresponding to (**d**) Pattern I, (**e**) Pattern VI and (**f**) Pattern VIII.

Since development of the radial glia/NPC is coordinated along the same spatiotemporal axis as the periventricular vascular plexus, we questioned whether the PVEC transcriptome qualitatively and/or quantitatively changes upon 48 h co-culture with NPC. ABEC co-cultured with NPC were used for comparison. Unsupervised principal component analysis (PCA) of the RNAseq data showed that cell type accounted for most of the variance between groups (PC1, 79.05%). Also, ABEC/ABEC + NPC clustered closer than PVEC/PVEC + NPC along the PC2 axis (7.68%), indicating a significantly greater impact of NPC co-culture on PVEC vs. ABEC transcriptomes (Fig. 3a).

Next, we performed supervised analysis to identify DEG in PVEC vs. ABEC monocultures or NPC co-cultures. A total of 6241 genes were differentially expressed in PVEC vs. ABEC, including 5842 genes in PVEC vs. ABEC monocultures, and 5712 in PVEC + NPC vs. ABEC + NPC. Of these genes, 529 were only differentially expressed in PVEC vs. ABEC at baseline, with 457 being exclusive to this comparison. On the other hand, 399 genes were only differentially expressed in PVEC + NPC vs. ABEC + NPC co-cultures, with 337 being exclusive to this comparison. Notably, 5313 genes were differentially expressed regardless of the culture condition, with 5053 being exclusively differentially expressed in PVEC vs. ABEC and PVEC + NPC vs. ABEC + NPC (Fig. 3b).

NPC co-culture resulted in robust changes in PVEC but not in ABEC transcriptome. There were 413 DEG in PVEC vs. PVEC + NPC, 224 were upregulated and 189 were downregulated. In contrast, just 15 genes were differentially expressed in ABEC vs. ABEC + NPC, 6 were upregulated and 9 were downregulated. Only 2 of the genes that were differentially expressed upon co-culture with NPC were common to PVEC and ABEC: lysophosphatidylcholine acyltransferase 2 (*Lpcat2*), was differentially expressed across all comparison groups (30-fold higher in PVEC vs. ABEC and in PVEC + NPC vs. ABEC + NPC, and threefold higher in PVEC + NPC vs. PVEC and in ABEC + NPC vs. ABEC) and synuclein alpha (*Snca*) was only differentially expressed when PVEC or ABEC were co-cultured with NPC (threefold higher in ABEC + NPC vs. ABEC and 2.5-fold higher in PVEC + NPC vs. PVEC) (Fig. 3b). Glucosaminyl (N-acetyl) transferase 1 (*Gcnt1*) was the only gene whose expression was exclusively changed in ABEC + NPC vs. ABEC (twofold higher). On the other hand, 30 genes were exclusively modified in PVEC + NPC vs. PVEC, including regulators of cell proliferation, such as the BCL2/adenovirus E1B interacting protein 3 (*Bnip3*) and cyclin dependent kinase inhibitor 1C (P57) (*Cdkn1c*), and SLC transporters such as the thiamine transporter *Slc35f3,* and the sodium bicarbonate co-transporter *Slc4a10* that regulates neuronal pH in the choroid plexus, all of which were upregulated by twofold to threefold after co-culture.

Of the 413 genes that were differentially expressed in PVEC vs. PVEC + NPC, 321 were also differentially expressed in PVEC vs. ABEC, as gauged by supervised analysis. Using the self-organizing map (SOM) unsupervised algorithm, we clustered these genes into patterns based on their expression profile in PVEC vs. ABEC monocultures, and on their directional change upon co-culture with NPC. Patterns I–IV include genes that were upregulated in PVEC upon NPC co-culture. In patterns I–III, this upregulation further amplified the physiologic differences between PVEC vs. ABEC (Fig. 3c), whereas in pattern IV it brought PVEC expression levels closer to that of ABEC. GO_bp enrichment analysis of these clusters, followed by data summarizing using REVIGO, identified biologic processes that were enriched in each pattern. These included “Regulation of anatomical structure size” in pattern I (Fig. 3d), and “Regulation of cellular component organization” in pattern II. Genes clustered in Patterns III and IV did not enrich for any specific GO_bp. Patterns V, VI and VII include genes whose basal expression levels were higher in PVEC vs. ABEC monocultures and were downregulated upon NPC co-culture to levels closer to that of ABEC. GO_bp enrichment analysis, followed by data summarizing using REVIGO of Pattern VI showed an enrichment for “Anatomical structure development” and “Regulation of cell communication” (Fig. 3e). Pattern VIII encompass genes whose expression was initially lower in PVEC vs. ABEC monocultures and slightly increased upon NPC co-culture, albeit remaining lower than ABEC’s levels. By GO_bp analysis and REVIGO summarizing, Pattern VIII enriched for “Positive regulation of innate immune response” and “Immune system process” (Fig. 3f).

### The top differentially expressed genes in PVEC, regardless of culture condition, capture the transcriptional signature of these cells

**Figure 4:**
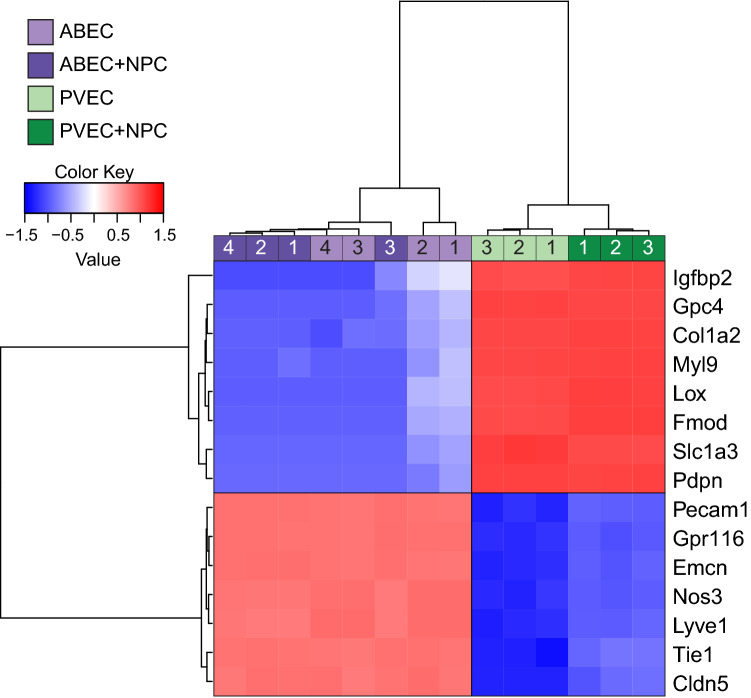
PVEC exhibit a distinct gene signature independent from culture condition. Hierarchical clustering and heatmap analysis of the top 15 DEG in PVEC (n = 6) vs. ABEC (n = 8). Gene expression is shown in a pseudocolor scale. Red indicates genes with higher expression and blue indicates genes with lower expression in PVEC vs. ABEC.

Heatmap analysis of the top DEG regardless of mono or co-culture conditions—proposed as the transcriptional signature of PVEC—is shown in Fig. 4. The most upregulated genes in PVEC/PVEC + NPC vs. ABEC/ABEC + NPC were insulin-like growth factor binding protein 2 (*Igfbp2*), *Lox*, fibromodulin (*Fmod*), *Myl9*, *Gpc4*, collagen, type I, alpha 2 (*Col1a2*), *Slc1a3* and *Pdpn*; the most downregulated genes were *Pecam1*, *Tie1*, *Nos3*, *Gpr116/Adgrf5*, *Emcn*, *Lyve1* and *Cldn5*. In addition to previously discussed EC markers (*Pecam1*, *Tie1* and *Nos3*), *Gpr116/Adgrf5*, an adhesion molecule that is also broadly expressed in EC, was 10,000-fold lower in PVEC/PVEC + NPC vs. ABEC/ABEC + NPC. The importance of *Gpr116/Adgrf5* expression in the brain endothelium is emphasized by the brain vascular leakage that occurs in *Gpr116/Adgrf5* EC-specific knockout mice^34^. Hierarchical clustering of the samples showed distinct separation between PVEC and PVEC + NPC. In contrast, ABEC vs. ABEC + NPC did not separate well (Fig. 4). Heatmap analysis of the data strongly corroborates the specific transcriptional signature associated with the developmental stage of PVEC.

### Pathway analysis of PVEC co-cultured with NPC ascertains an overall decrease in complement and increase in WNT/β-catenin related transcripts

**Figure 5:**
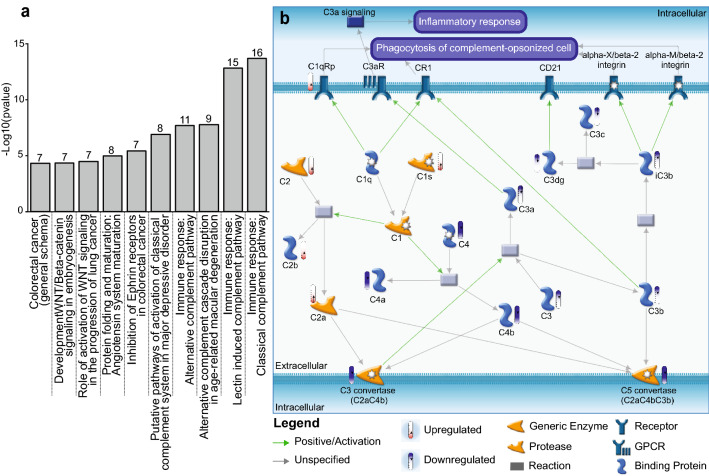
Canonical pathway enrichment analysis identifies immune response classical complement pathway as the ones most enriched in PVEC co-cultured with NPC. (**a**) Canonical pathway enrichment using the Metacore from Clarivate Analytics identified the top 10 most enriched pathways in PVEC vs. PVEC + NPC. (**b**) Depiction of the top most modified pathway, “Immune response: Classical complement pathway”. The legend included in the figure decodes the colored shapes and arrows used in the picture. Colored bars close to each protein name indicates whether the DEG was upregulated (red) or down-regulated (blue) upon co-culture of PVEC with NPC.

In order to better understand the effect of NPC co-culture on PVEC, we performed canonical pathway enrichment analysis on DEG. Among the top ten most modified pathways, five were related to the immune system response/complement, namely classical complement (16 genes modified in our dataset out of 53 in database), lectin-induced complement (15 out of 50), and alternative complement (9 out of 31) (Fig. 5a). Remarkably, the pathway with highest enrichment score in PVEC + NPC i.e. “Immune response: Classical complement pathway” included key components of the complement system whose transcript levels were significantly decreased upon co-culture. For instance, a decrease in complement component 3 (C3, 4.7-fold) and C4 (31.5-fold) implies lower levels of the C3 and C5 convertase complexes (Fig. 5b). Similar to our baseline PVEC vs. ABEC data, 2 of the 10 enriched pathways were related to WNT signaling, namely the “role of activation of WNT in the progression of lung cancer” (7 out of 41), and “development: WNT/β catenin signaling in embryogenesis” (7 of 43) (Fig. 5a).

### Network analysis emphasizes the maintenance of stemness as a feature of PVEC

We previously reported that co-culturing PVEC with NPC delays expression of β-tubulin, a marker of stemness in NPC^4^. To better characterize this observation, we performed a network analysis of PVEC vs. PVEC + NPC transcriptomes and identified ‘Oct-3/4, NANOG, Neuronatin, TRIL’ as one of the top modulated networks (Supplementary Figure 4). A 100-fold increase in mRNA levels of *Pou5f1* that encodes for Oct-3/4, a transcription factor that promotes pluripotency and self-renewal of undifferentiated cells^35^, was the most salient change in this network. Also, an increase in the transcription factor *Nanog* (2.4-fold) and a decrease in neuronatin (*Nnat,* twofold), that directs cells into the neural lineage^36^, strongly validates our proposed hypothesis—PVEC and NPC cross-talk to maintain their respective early-stage of differentiation. In addition to influencing stemness, higher Oct3/4 transcripts in PVEC co-culture were associated with 2–fourfold lower levels of pro-inflammatory TLR4 interactor with leucine-rich repeats (*Tril*). *Tril* is highly expressed in the brain, specifically in glial cells where its knockdown attenuates cytokine production^37^. These latter results further highlight the combined anti-inflammatory and maintenance of stemness phenotype of PVEC.

## Discussion

We previously showed that one of the functions of PVEC is to maintain the proliferative capacity and the progenitor status of NPC^4^. To better characterize these cells, we compared their basal transcriptome to that of adult brain EC. Our data demonstrate that PVEC and ABEC transcriptomes are strikingly distinct at baseline (> 5000 genes), reflecting the mature state of ABEC and their barrier properties (high expression of *Tie1, Pecam1, Nos3*, and *Cldn5*) vs. the immature state of PVEC. PVEC immaturity is evidenced by their expression of genes usually associated with periventricular NPC, namely *S1pr3, Igfb2, Igfbp5, Slc1a3* and *Gpc4*^31,38–40^. SIPR3 drives S1P-induced proliferation of NPC^38^, IGFBP2 and IGFBP5 promote NPC proliferation and self-renewal, whilst precluding their differentiation^39^, and *Slc1a3* contributes to NPC proliferation through glutamate transport^41^. Heparan sulphate proteoglycan, Glypican4 (GPC4) modulates FGF signal transduction, which is required for early dorsoventral forebrain patterning^40^. This agrees with *Fgf2* transcripts being significantly higher in PVEC vs. ABEC.

PVEC also expressed higher levels of glucose transporters (*Slc2a3* and *Slc2a13*) and glutamate transporters (*Slc1a3, Slc1a2, Slc1a6* and *Slc38a3*). The brain receives about 20% of total body glucose and uses 80% of this energy to maintain the homeostasis of the glutamate/GABA glutamine cycle. While glutamate is important for synaptic function, its extracellular levels must be tightly controlled to avoid excitotoxicity^42^. Lack of the brain-specific *Slc2a3* (*Glut3*) has profound effects on neural development^30^. Accordingly, its heightened expression in PVEC highlights the unique ability of these cells to promote brain development. The abundance of glutamate transporters in PVEC may have different implications, either to provide the substrate for NPC proliferation and synthesize GABA to generate GABAergic neurons, or to increase the brain-to-blood efflux of glutamate (*Slc38a3*) in order to limit excitotoxicity.

ABC transporters, including *Abca9, Abca8a, Abca8b* and *Abcg4*, all involved in lipid transport and metabolism were also higher in PVEC. This could be an important feature of PVEC since regulation of cholesterol homeostasis in the CNS relies on de novo cholesterol synthesis and efficient lipid transport and recycling within the brain^43^. Consistent with the latter, PVEC also expressed significantly higher levels of Mfsd2a (147-fold) than ABEC, an omega-3 fatty acid transporter that is essential for brain development^44^.

Other genes whose expression was higher in PVEC, namely *Lox*, *Fmod* and *Col1a2*, are involved in the formation of ECM. The extracellular enzyme Lysyl oxidase (LOX), that deaminates peptidyl lysyl residues to promote cross-linking of fibrillar collagens and elastin, activates AKT in EC to increase VEGF expression and promote angiogenesis^45^. This agrees with significantly higher *Vegf* transcripts in PVEC vs. ABEC. The ECM protein Fibromodulin (FMOD) participates in the assembly of collagen fibers. Since overexpression of FMOD can reprogram cells to express pluripotency markers^46^, we surmise that its heightened expression in PVEC maintains the neurovascular niche. Furthermore, Collagen I triggers EC to assume a spindle-shaped morphology and to align into solid cord-like structures that resemble the pre-capillary cords of embryonic angiogenesis^47^. Hence, higher levels of Collagen I in PVEC is in line with these cells’ ability to regulate angiogenesis and neurovascular patterning in the embryonic brain.

By GO analysis of DEG in PVEC vs. ABEC. “Nervous system development” and related categories were the most enriched, mostly with genes that were higher in PVEC vs. ABEC. This result concurs with neural development as the dominant program that characterizes this key function of PVEC.

On analyzing differentially expressed pathways, HIF-1A topped the list. This corresponds with embryo development occurring in a hypoxic environment where many of the HIF-1A targets are coopted to fulfill the requirements of a rapidly growing brain^48^. Although *Hif-1a* mRNA levels were lower in PVEC vs. ABEC, transcript levels of many of its direct target genes were significantly higher, suggesting its activity was increased in these cells. For example, *Cxcr4*, a HIF-1A target gene expressed in rapidly dividing Nestin positive NPC, had its mRNA levels > 400-fold higher in PVEC vs. ABEC^49^, so were transcript levels of the HIF1-A pro-angiogenic targets *Vegfa, Fgf2*, *Thrombospondin1*, and *Angiopoietin2*^50^.

When we restricted our analysis to DEG that were significantly higher in PVEC, the top enriched pathway was WNT signaling. WNT/β-catenin signaling prevents premature differentiation of NPC to neurons^51^. Higher expression of members of this pathway in PVEC corroborates our previous results showing that PVEC, but not ABEC, delay NPC differentiation^4^. Indeed, expression levels of the LEF-1 transcription factor, a positive regulator of NPC proliferation, was 64-fold higher in PVEC vs. ABEC. WNT signaling also positively regulates angiogenesis in the brain and contributes to BBB formation and integrity^52,53^. WNT ligands are expressed by NPC in the ventricular region during the same epoch as cerebral angiogenesis. Notably, transcript levels of the WNT ligands, *Wnt7a* and *Wnt7b*, and of their receptors (Frizzled) were significantly higher in PVEC vs. ABEC. This suggests that PVEC act in an autocrine and paracrine manner to maintain the proliferative status of the NVU.

When we restricted our analysis to DEG that were significantly lower in PVEC, the top enriched pathway was “Immune response IFNα/β signaling via JAK/STAT”, a novel observation during brain development that dovetails with data indicating that mounting an antiviral response causes differentiation of pluripotent stem cells^54^. Immune activation and secondary upregulation of chemokines and cytokines negatively regulate NPC proliferation and drives their differentiation into the neuronal and astroglial lineages in a STAT-dependent manner. Our results show that 5 of the 6 STAT, i.e. *Stat1*, 2, 4, 5 and 6 were significantly lower in PVEC vs. ABEC. This is in keeping with expression levels of most these STAT being characteristically low (1, 4 and 5) or even undetectable (STAT6) in the embryonic mouse brain^55,56^. Importantly, transcript levels of a number of downstream targets of these STATs were also significantly lower in PVEC vs. ABEC. These include pro-apoptotic XAF1 and Caspase-8^57,58^, RIG-G, a key mediator of the IFNα/β anti-proliferative effect^59^, and IRF7 that drives stem cells to mesodermal differentiation. In fact, expression of the pluripotency genes KLF4, SOX2 and OCT4 is totally irreconcilable with the transcriptional program of IRF7^54^. Altogether, reduced expression of these 4 genes in PVEC showcases their stemness, proliferative capacity, and resistance to apoptotic stimuli. *Stat3*, is the only STAT member whose expression is threefold higher in PVEC vs. ABEC. STAT3 activation maintains the undifferentiated state of SVZ progenitors^60^ while its loss decreases the number of Nestin positive NPCs in the brain^61^. Consequently, higher levels of STAT3 in PVEC serves the same purpose as lower expression of the other STATs i.e. maintaining stemness and boosting proliferation.

Although differences between PVEC and ABEC transcriptomes at baseline far exceeded any effects of NPC co-culture, the PVEC, but not the ABEC, transcriptome still significantly changed by co-culture (413 vs. 15 genes), highlighting their unique partnership with NPC. Decreased expression of complement immune response genes was exclusively noted in PVEC co-cultured with NPC. This is a novel observation whose biologic consequences still need to be investigated. The only known non-immune function of complement in the brain is to regulate normal postnatal synaptic pruning and brain wiring^62^.

In conclusion, our data characterizes for the first time the gene signature of PVEC and their distinct partnership with NPC. The identity of these cells underpins their unique role in promoting brain development and maintaining a low inflammatory environment that supports a healthy neurovascular niche. Although we acknowledge that this study may still be limited by some changes that may occur following EC culturing and serial passaging, it offers a road map for additional studies aimed at expanding PVEC characterization by analyzing their transcriptome in their anatomic context and exploring their neuro-regenerative potential in different brain pathologies.

## Materials and methods

### Ethical consideration

All mice use was in strict compliance with current United States government regulations concerning the care and use of laboratory animals. Animal protocols were approved by the Beth Israel Deaconess Medical Center Institutional Animal Care and Use Committee. All mice were anesthetized with isoflurane during procedures and provided with appropriate pain relief.

### Cell lines

Mouse primary ABEC derived from C57BL/6 were obtained from CellBiologics (Chicago, IL) and grown in DMEM-KO medium supplemented with 10% fetal bovine serum (FBS), 10% horse serum, 100 µg Endothelial Mitogen (Biomedical Technologies, Stoughton, MA), 4 mM l-Glutamine and 1% penicillin/streptomycin.

The neural progenitor NE-4C cell line (NPC) was obtained from American Type Culture Collection (Manassas, VA), and expanded for use in this study. This cell line maintains progenitor status, as evaluated by specific markers, after multiple passages^2^. NE-4 cells were cultured in DMEM-KO media supplemented with 10% FBS, 4 mM l-Glutamine, 1% Penicillin/Streptomycin.

### Isolation of PVEC from embryonic mice brain

At E15-16 the pial vessels from male and female C57BL/6 mice (Charles River Laboratories, Wilmington, MA) were removed^63^. The remaining telencephalon brain containing the periventricular vessels was dissociated into single cells as described^4^, and plated on a Type I rat tail collagen (BD Bioscience, San Jose, CA) coated flask containing DMEM-KO medium with 10% FBS (Fisher Scientific, Pittsburgh, PA), 10% Horse Serum, 100 μg Endothelial Mitogen (Biomedical Technologies, Stoughton, MA), 4 mM l-Glutamine, and 1% Penicillin/Streptomycin. After approximately 5 days, endothelial cells were purified using the EasySep mouse phycoerythrin (PE) positive selection kit conjugated to CD31 (Stemcell Tech, Vancouver, Canada). Cells labeled with anti-CD31, were bound to dextran coated magnetic particles by tetrameric antibody complexes recognizing PE and dextran, as per the manufacturer recommendations. Only those CD31 positive cells that bound to the magnetic beads were further cultured and used at passage 5 in all subsequent experiments. Flow cytometry was used to confirm endothelial-specificity using *bona fide* EC markers as described^4^ (Supplementary material and methods and Supplementary Figure 1).

### RNA extraction

In order to compare baseline gene expression between PVEC and ABEC, cells were grown as monocultures for 48 h before RNA extraction using the RNeasy Mini Kit (Qiagen, Germantown, MD). To understand the NPC effect on global gene expression, PVEC and ABEC (3 × 10^4^ EC /cm^2^) were seeded in the lower chamber of a 24-transwell plate and 1 × 10^4^ NPC/cm^2^ were added to the upper chamber. We kept the co-culture for 48 h in 0.1 mL and 0.5 mL complete medium in the upper and lower chambers, respectively. After this time, RNA was extracted from the EC seeded in the lower chamber.

### Transcriptional profiling

Double-stranded cDNA sequencing libraries were generated using the Illumina TruSeq kit per the manufacturer’s protocol. High quality libraries were sequenced on an Illumina HiSeq 2000 (Illumina, Inc., San Diego, CA). To achieve comprehensive coverage for each sample, we generated ~ 30–35 million paired end reads. Raw sequencing was pre-processed, quality checked, aligned to mouse genome, and unique numbers of reads counted. The read count-based expression data was normalized using the voom approach that estimates the mean–variance relationship of the log-transformed transcript counts data to generate a precision weight for each expressed transcript^64^. Differentially expressed transcripts were identified from the normalized dataset using Metaboanalyst and Metacore analysis workflow (Clarivate Analytics ver. 6.11, build 41105, GeneGo, Thomson Reuters, USA), based on absolute fold change (FC) and multiple test-corrected p-values based on FDR. Transcripts were considered significantly differentially expressed if p-values were < 0.05 and absolute fold change (FC) was > 2.

### Self organizing map (SOM) clustering

We used the SOM clustering technique to identify group-dependent patterns of DEG in PVEC vs ABEC and PVEC vs PVEC + NPC^65^. SOM allows the grouping of gene expression patterns into an imposed structure in which adjacent clusters are related, thereby identifying sets of samples that follow certain expression patterns across groups. We performed sample-based SOM clustering (som package in R) using Pearson correlation coefficient-based distance metrics, which resulted in six sample clusters with distinct expression profiles.

### Statistical analysis

#### Unsupervised analysis

Principal component analysis (PCA) and hierarchical clustering were used to perform unsupervised analysis on normalized and preprocessed data. Analysis were made in R and GenePattern^66^.

#### Supervised analysis

MetaCore from Clarivate Analytics was used to perform enrichment analysis in order to determine the functional significance of DEG. We identified the top modified pathways and networks in PVEC vs. ABEC, PVEC + NPC vs. ABEC + NPC, PVEC vs. PVEC + NPC and ABEC vs. ABEC + NPC. The GO_bp results were subjected to a REduce and Visualize Gene Ontology tool (REVIGO)^67^.

## Supplementary information

Supplementary Information.
