# Supplementary material for: Embryonic periventricular endothelial cells demonstrate a unique pro-neurodevelopment and anti-inflammatory gene signature
Source: Sci Rep. 2020 Nov 23;10:20393. doi: 10.1038/s41598-020-77297-3 (PMC7683543; doi:10.1038/s41598-020-77297-3)
Supplement: Supplementary file 1 — Supplementary Information. [file 41598_2020_77297_MOESM1_ESM.pdf]

**Title: Embryonic periventricular endothelial cells demonstrate a unique pro-neurodevelopment and anti-inflammatory gene signature**

**Authors:** Franciele Cristina Kipper[1,5,7], Cleide Angolano[2,5,7], Ravi Vissapragada[1,8], Mauricio A. Contreras[3], Justin Moore[1,7], Manoj Bhasin[6], Christiane Ferran[2,3,7,#] & Ajith J. Thomas[1,7,#,*]

1. Division of Neurosurgery, Beth Israel Deaconess Medical Center, Boston, MA, 02215, USA
2. Division of Vascular and Endovascular Surgery, Department of Surgery, Beth Israel Deaconess Medical Center, Boston, MA, 02215, USA
3. Division of Vascular Surgery, Department of Surgery, Beth Israel Deaconess Medical Center, MA, 02215, USA
4. Division of Nephrology, Department of Medicine, Beth Israel Deaconess Medical Center, MA, 02215, USA
5. Center for Vascular Biology Research, Beth Israel Deaconess Medical Center, Boston, MA, 02215, USA
6. BIDMC Genomics, Proteomics, Bioinformatics and Systems Biology Centre, Beth Israel Deaconess Medical Center, Boston, MA, 02215, USA
7. Harvard Medical School, Boston, MA, 02215, USA
8. Discipline of Surgery, College of Medicine and Public Health, Flinders University, Adelaide, SA, 5042 Australia

# Joint last authorship

* Corresponding author. E-mail address: athomas6@bidmc.harvard.edu (Ajith Thomas)

## SUPPLEMENTAL MATERIAL AND METHODS

### Flow cytometry

After cell isolation of PVEC with EasySep mouse phycoerythrin (PE) positive selection kit conjugated to CD31 (Stemcell Tech, Vancouver, Canada), the purity of CD31-PE cells was evaluated by flow cytometry. The acquisition and the analysis was performed on a FACSAria III cell sorter. No fixatives or permeabilization agents were used.

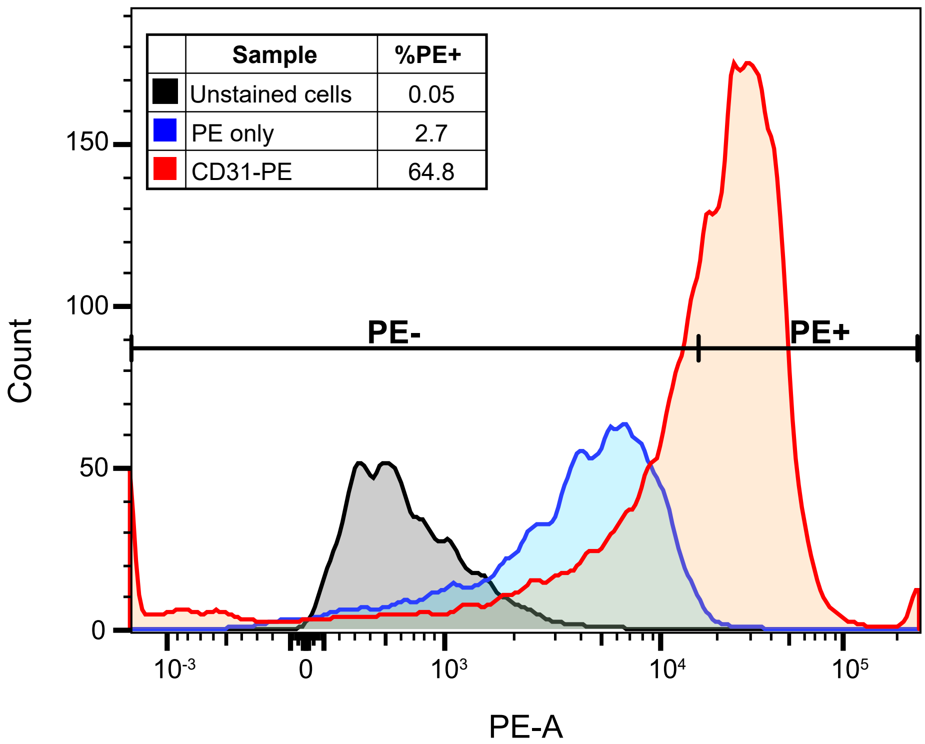


**Supplementary Figure 1: Characterization of PVEC based on CD31 staining.** PVEC were isolated and dissociated from the mouse brain telencephalon (E 15.5) and grown until confluence. Following this, the endothelial population was purified using the EasySep mouse PE selection kit for magnetic sorting. Purity of CD31+ cells was evaluated by flow cytometry before passaging. Histograms shows that more than 60% of these cells were considered endothelial cells based on CD31 positivity in contrast to 0% of unlabeled cells and 2.7% of PE-conjugated isotype control (Rat IgG2a, κ).

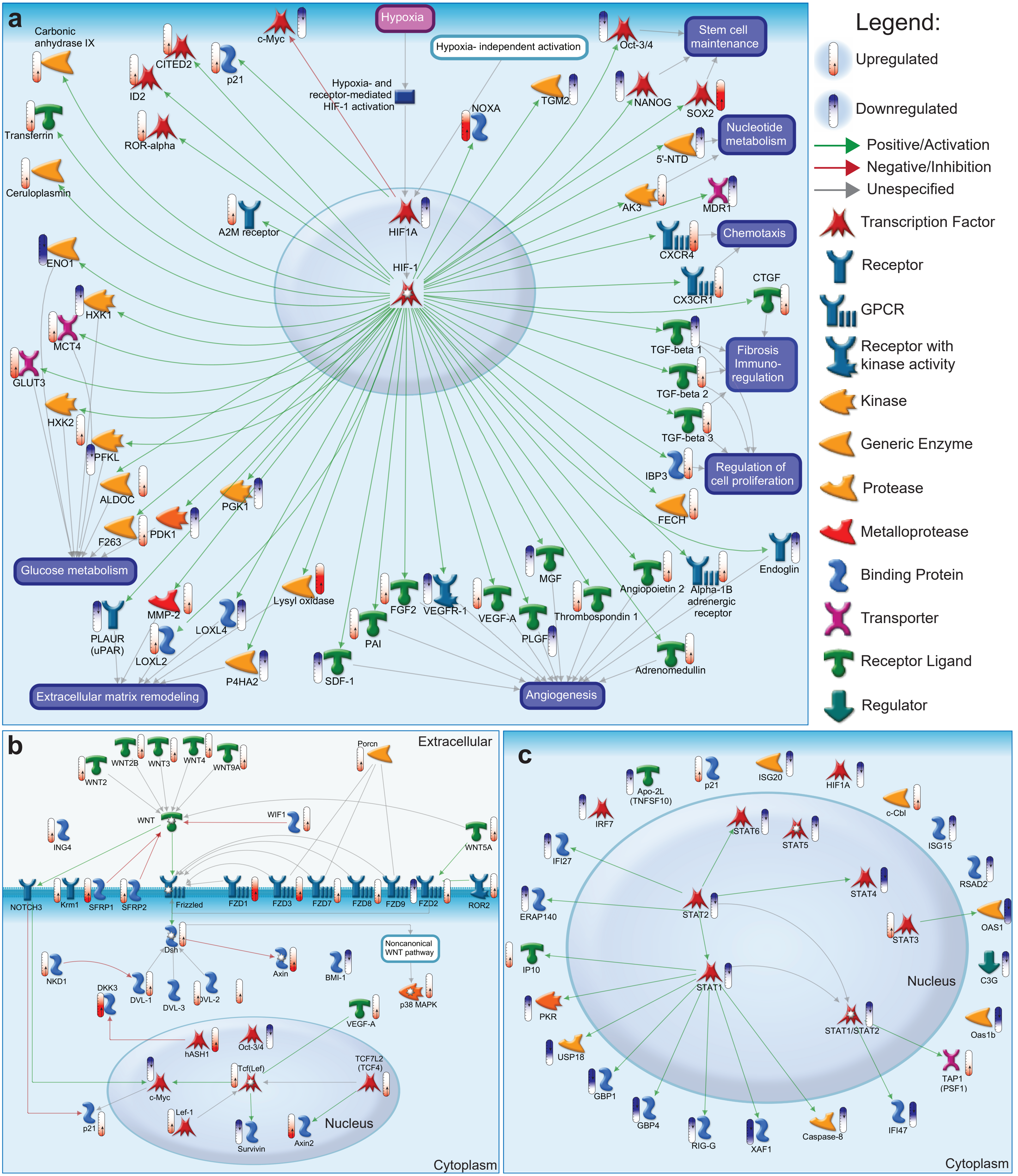


**Supplementary Figure 2: "Transcription: HIF-1 targets" is the top differentially enriched pathway in PVEC vs. ABEC.** DEG involved in the WNT signaling pathway were predominantly higher while those related to the JAK/STAT transcription factors pathway were predominantly lower in PVEC vs. ABEC  Schematic representation of A) the "Transcription: HIF-1 targets" pathway depicting the 54 differentially expressed genes. B) "Role of activation of WNT signaling in the progression of lung cancer" pathway highlights the 36 most DEG, and C) "Immune response: IFN-alpha/beta signaling via JAK/STAT" pathway highlights the 25 most DEG. The legend included in the figure decodes the colored shapes and arrows used in the picture. Colored bars adjacent to each protein's name indicates whether expression of its corresponding gene was higher (red) or lower (blue), in PVEC vs. ABEC.

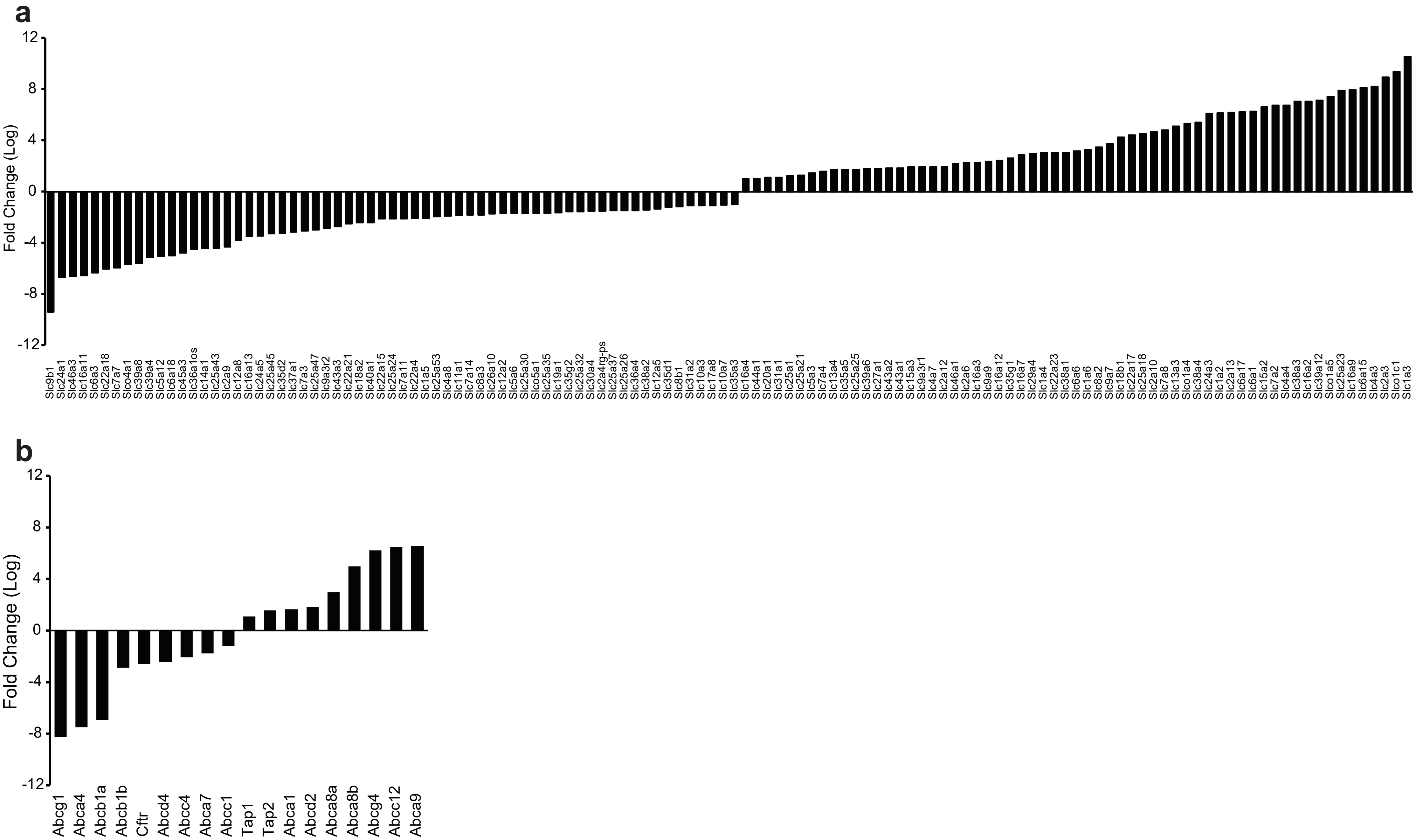


**Supplementary Figure 3: Differentially expressed SLC and ABC transporters in PVEC vs ABEC.** Waterfall depiction of A) 124 SLC transcripts (61 higher and 63 lower), and of B) 18 ABC transporters (9 higher and 9 lower) that were differentially expressed in PVEC vs. ABEC.

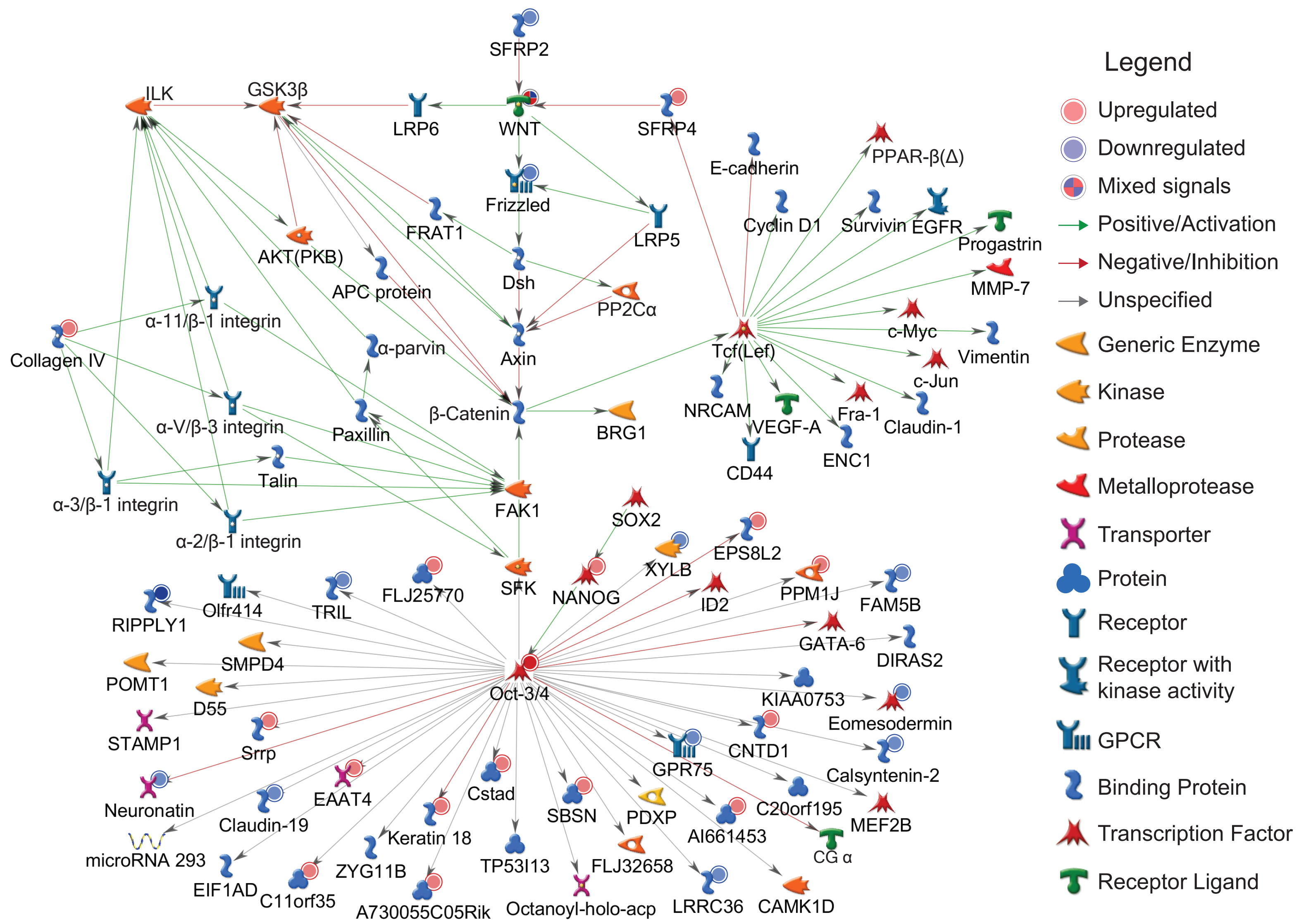


**Supplementary Figure 4: Network analysis features increased PVEC stemness upon co-culture with NPC.** Merge of the top 2 most modified networks in PVEC vs. PVEC+NPC identify Oct-3/4 as a central hub of the network. Networks were built based on DEG using the Metacore software. The legend included in the figure decodes the colored shapes and arrows used in the picture. Colored circles close to each protein name indicates whether the DEG was upregulated (red) or down-regulated (blue) when PVEC were co-cultured with NPC.
